# Prevalence and Risk Factors for Musculoskeletal Pain when Running During Pregnancy: A Survey of 3102 Women

**DOI:** 10.1007/s40279-024-01994-6

**Published:** 2024-02-06

**Authors:** Hannah E. Wyatt, Kelly Sheerin, Patria A. Hume, Kim Hébert-Losier

**Affiliations:** 1 Faculty of Health, University of Canterbury, Christchurch, New Zealand; 2 Sports Performance Research Institute New Zealand, Auckland University of Technology, Auckland, New Zealand; 3 Auckland Bioengineering Institute, The University of Auckland, Auckland, New Zealand; 4 Mindaroo Tech & Policy Lab, Law School, The University of Western Australia, Perth, Australia; 5 Division of Health, Engineering, Computing and Science, Te Huataki Waiora School of Health University of Waikato, Tauranga, New Zealand

## Abstract

**Background:**

Musculoskeletal pain while running is a concern to women during pregnancy and can lead to running cessation. To support women who wish to run during pregnancy, it is essential to understand the sites, severities and personal risk factors associated with musculoskeletal pain.

**Objective:**

The aim was to investigate prevalence and risk factors for musculoskeletal pain when running during pregnancy.

**Methods:**

An online survey was completed by women who had a child in the past 5 years and ran prior to and during pregnancy. Pain frequency informed prevalence rates by body site, and logistic regression odds ratios (ORs) and 95% confidence intervals were calculated.

**Results:**

A total of 3102 women of 23 ethnicities from 25 countries completed the survey. Women were 22–52 years old when they gave birth and ran 2–129 km/week during the 0.5–35 years before the birth of their youngest child. Women ran significantly less distance and less often during pregnancy than before pregnancy. Most women (86%) experienced pain while running during pregnancy (59% pelvis/sacroiliac joint, 52% lower back, 51% abdomen, 44% breast, 40% hip). The highest prevalence of severe-to-worst pain was at the pelvis/sacroiliac joint (9%). Women at greatest risk of pain while running during pregnancy had a previous injury (OR = 3.44) or were older (OR = 1.04). Women with a previous child were less likely to experience breast pain (OR = 0.76) than those running during their first pregnancy.

**Conclusion:**

Healthcare practices to reduce pain should focus on regions of greatest musculoskeletal change during pregnancy, specifically the pelvis, lower back and abdomen. Efforts to support women to run for longer throughout pregnancy should focus on pain at the pelvis and breasts.

**Supplementary Information:**

The online version contains supplementary material available at 10.1007/s40279-024-01994-6.

## Key Points

**Table:** 

Eighty-six per cent of women reported pain while running during pregnancy, most commonly at the pelvis/sacroiliac joint, lower back, abdomen, breast and hip.
Women who had a previous child were more likely to experience pelvic/sacroiliac joint, hip, knee and lower back pain, but less likely to experience breast pain than those in their first pregnancy.
Pain experienced at the pelvis and breasts were the greatest musculoskeletal barriers to continuation of running during pregnancy; therefore, pain prevention strategies should prioritise these sites.

## Introduction

Physical activity is beneficial during pregnancy; however, less than a third of pregnant women^1^ meet the minimum recommendations of 150 min of moderate-intensity activity per week [2, 3]. Enabling pregnant women with uncomplicated pregnancies to continue participating in physical activity can enhance their health and lower the risk of complications during childbirth [4]. Women who exercise earlier in pregnancy and maintain higher activity levels throughout pregnancy report less pregnancy-related discomfort and are more likely to return to postpartum exercise, such as running [5].

Running is a popular and accessible form of exercise that is considered safe for women who ran before pregnancy [2]. Many women aim to continue running during pregnancy; however, participation has been documented to decrease as pregnancy progresses, with only 31% of competitive runners continuing to run during their third trimester [6]. Common reasons for running cessation include experiences of musculoskeletal pain in the pelvis, hip, back and abdominal regions [7]. Alongside hormonal [8], laxity [9] and other physiological changes [10], gait alterations during pregnancy have the potential to influence the type and location of pain experienced by pregnant women [11]. The prevalence and distribution of pain across body sites is yet to be explored for women running during pregnancy.

Musculoskeletal pain is integral to running-related injury definitions [12], which dominate the focus of nulliparous running-related research. Running-related injuries in the nulliparous population predominantly occur at the lower limbs [13–16]; however, the definition of running-related injuries does not typically account for pain experienced within torso or upper-body regions [12]. Sites such as the lower back, pelvis, abdomen and breasts have been highlighted as prominent regions of concern for women during pregnancy [7, 17, 18]; therefore, consideration of general musculoskeletal pain for women who run during pregnancy is warranted. Given the close alignment between pain (a symptom) and injury (a diagnosis), insights from running-related injuries in nulliparous females may direct initial investigations of risk factors for running-related pain during pregnancy.

Within nulliparous populations, it is well understood that previous injury is a key running-related injury risk factor [19]. Older age [15, 20], running less than once weekly [15] and having a greater body mass index [20, 21] have been identified as additional running-related injury risk factors for females. Knowledge of prevalence and risk factors is an important first step in developing and implementing pain prevention strategies [22–24] that aim to support continued engagement in activities such as running. It is common for quality of life to be negatively impacted by experiences of pain, with consequences that can extend beyond the physical sensations; for example, those who experience musculoskeletal pain have been found to show more catastrophising and fear-avoidance beliefs [25]. Understanding pain patterns in pregnant women routinely engaging in running is therefore an important step in mitigating musculoskeletal risk, enhancing physical activity enjoyment and health-related quality of life.

### Aim and Research Questions

The aim of the study was to develop insight into the prevalence and risk factors for musculoskeletal pain when running during pregnancy. Questions of interest to the research team were:

1. How do running habits for women change from pre- to during pregnancy?
2. What is the prevalence and severity of pain while running during pregnancy?
3. To what extent do personal and pre-pregnancy factors affect the odds of experiencing pain while running during pregnancy?

## Materials and Methods

### Experimental Design

As part of this descriptive cross-sectional study, a convenience sample of women who varied by age, ethnicity and number of children was surveyed.

### Participants

The inclusion criteria required women to be over 18 years old and to have regularly run at least once per week for a minimum of 20 min before their most recent pregnancy and during any part of their pregnancy. Women needed to have had a child within 5 years of the survey response date.

### Survey Development

A multidisciplinary team of four academics, two physiotherapists, three biomechanists, a midwife and a pregnant woman who ran during pregnancy developed the survey. The survey was tested for usability and technical functionality by the team. A wider group of four women who had run while pregnant then assessed the survey readability prior to final confirmation. Ethics approval was gained from the Auckland University of Technology Ethics Committee (#21/410) and the parkrun Research Board.

### Data Collection

Details of the estimated time to complete the survey, data storage, investigator details and purpose of the study were outlined in the participant information sheet (see the electronic supplementary material)*.* The online survey was anonymised; the participants indicated they had read and understood the study information, and considered they met all inclusion criteria before completing the survey.

The final survey was advertised internationally on social media (Twitter, Instagram and Facebook) between December 2021 and March 2022. The advertisement is included in the electronic supplementary material. An invitation to participate in the survey was also distributed via email by the United Kingdom (UK)-based *parkrun* organisation to their participant database in March 2022. The online survey was administered via Qualtrics software (Qualtrics XM, Provo, UT) and was accessed via a direct Qualtrics link. The survey was open-access, i.e. not password restricted, and responses were voluntary. The survey was open from 2 December 2021 until 14 March 2022 and was available in English only. No incentives were provided for the completion of the survey.

Questions related to the 6 months prior to pregnancy (pre-pregnancy) and during the pregnancy of the woman’s youngest child. Question order followed logical chronological progression (i.e. pre-pregnancy, during pregnancy and postpartum) and was consistent for all participants; however, conditional questioning was used based on individual question responses. The number of questions displayed per page ranged from one to eight and covered 11 pages for all questions. Respondents were given the option of reviewing and changing their answers through use of a ‘back’ button.

Survey questions captured demographic information, running habits (pre- and during pregnancy), pre-pregnancy injury and during-pregnancy physical pain experiences. For ease of recall, women were able to answer running distance-based questions in either miles or kilometres; all were converted to kilometres for consistency of reporting (questions are supplied in the electronic supplementary material).

Pre-pregnancy running experience categories were defined as ‘novice’ (less than 6 months of running at least once per week), ‘recreational’ (between 6 months and 3 years of running at least once per week) and ‘experienced’ (more than 3 years of running at least once per week) [26]. During-pregnancy running distance and frequency were grouped by trimester (trimester 1 = 0–13 weeks gestation; trimester 2 = 14–27 weeks gestation; trimester 3 = 28 to > 40 weeks gestation).

Body sites were categorised as follows: breast, abdominal, lower back, pelvis/sacroiliac joint (SIJ), hip, thigh, knee, calf, ankle and foot. Pain was categorised as 'no pain’, ‘mild’, ‘moderate’, ‘severe’, ‘very severe’ or ‘worst pain possible’.

### Data Analyses

Participation rate was calculated as the percentage of consented respondents who answered survey questions beyond the demographic information. Response completeness rate was calculated from Qualtrics metrics for all participants. Responses to demographic questions were descriptively analysed using frequencies (percentages), means, standard deviations (± SD) and ranges. Text responses were coded using NVivo qualitative data analysis software (v20, QRS International, MA, USA) and counted to inform pre-pregnancy musculoskeletal injury prevalence. Prevalence rates were calculated for each body site as a percentage of those women who had a pre-pregnancy injury. Body site pregnancy pain prevalence rates were calculated as a percentage of women who reported pain at any body site during pregnancy.

### Statistical Analyses

To compare during-pregnancy running distances and frequencies (trimesters 1, 2 and 3) to pre-pregnancy running, Wilcoxon signed-rank tests were conducted following tests of normality. Binary logistic regression analyses were conducted to inform musculoskeletal pain aetiology during pregnancy. The dependent variable was pain (yes/no) at each of the ten body sites. Discrete predictor variables were ‘any previous recurring pre-pregnancy injury’ (yes/no) or ‘previous children’ (1 or > 1 child). Continuous predictor variables were ‘maternal age at childbirth’ (years), ‘time of running cessation’ (weeks of gestation), ‘pre-pregnancy running experience’ (years), ‘running distance’ (km) and ‘running frequency’ (times/week). A criterion of *p* < 0.05 defined statistical significance. Predictors of pain at different sites were reported as odds ratio (OR) and 95% confidence interval (CI). All statistical analyses were conducted in SPSS software (v29.0, IBM Corp., Armonk, NY, USA).

## Results

Of the original 3554 women who provided study consent, 112 women did not meet all inclusion criteria and a further 340 women who completed demographic information did not complete any survey questions. These respondents were excluded from analyses, leaving data for 3102 women (87% participation rate). Survey completeness was 99.1 ± 5.4% (59–100%). Question responses were reviewed for unreasonable outliers, which were removed (< 0.1% of all question responses). Women were not excluded from analyses if some question responses were missing; hence, not all 3102 women informed every analysis. The number of women informing each analysis are included within results where relevant.

### Demographics

Of the 3102 women who responded, the average postpartum status (difference between age when completing the survey and age at birth of youngest child) was 1.9 ± 1.4 years. Only 1% of women reported pregnancies involving multiple babies, and 52.6% reported on their first pregnancy. More than 23 ethnicities were represented (see Supplementary Table A in the electronic supplementary material); most women were of British ethnicity (62.2%), followed by Australian (11.8%). Respondents were from 25 countries (see Supplementary Table B)*.*

### Running Habits

Most women were experienced runners (72.5%), with 25.3% recreational and 1.3% novice. Running experience ranged from < 3 years (11.9%) to > 20 years (3.5%), with a cohort mean of 8.6 ± 6.0 (range 0.5–35) years. On average, women ran 3 times/week for a total of 23.5 ± 18.0 km before pregnancy (Table 1). Running distances and frequencies decreased as pregnancy progressed and were significantly lower (*p* < 0.001) than pre-pregnancy at trimester 1 (mean decrease of 5.3 km and 0.3 times/week), trimester 2 (mean decrease of 8.5 km and 0.6 times/week) and trimester 3 (mean decrease of 10.6 km and 0.8 times/week) (Table 1). 7.8% of women stopped running during trimester 1, 41.6% in trimester 2, 43.3% in trimester 3 (before 1 week of birth) and 7.2% ran within 1 week of giving birth.

**Table 1:** Demographic, pre-pregnancy and during-pregnancy metrics for the 3102 women

	Mean ± SD	Range (min–max)
Age at completing the survey (years)	36.1 ± 4.1	24–54
Age at birth of youngest child (years)	34.0 ± 3.9	22–52
Gestation at birth (weeks)	39.2 ± 1.9	24–42.5
Number of children	2 ± 1	1–7
Pre-pregnancy running experience (years) (3102 women)	8.6 ± 6.0	0.5–35
Pre-pregnancy run distance (km/week) (3010 women)	23.5 ± 18.0	2–129
Pre-pregnancy run frequency (times/week) (3098 women)	3.2 ± 1.3	1–9
Running distance (km)		
Trimester 1 (2832 women)	18.2 ± 14.9*	1–113
Trimester 2 (2553 women)	15.0 ± 12.8*	1–100
Trimester 3 (1299 women)	12.9 ± 11.2*	1–90
Running frequency (times/week)		
Trimester 1 (2897 women)	2.9 ± 1.3*	0.5–13
Trimester 2 (2600 women)	2.6 ± 1.3*	0.5–14
Trimester 3 (1325 women)	2.3 ± 1.4*	0.5–12

*Significantly lower than pre-pregnancy, *p* < 0.05

### During-Pregnancy Pain

**Fig. 1:**
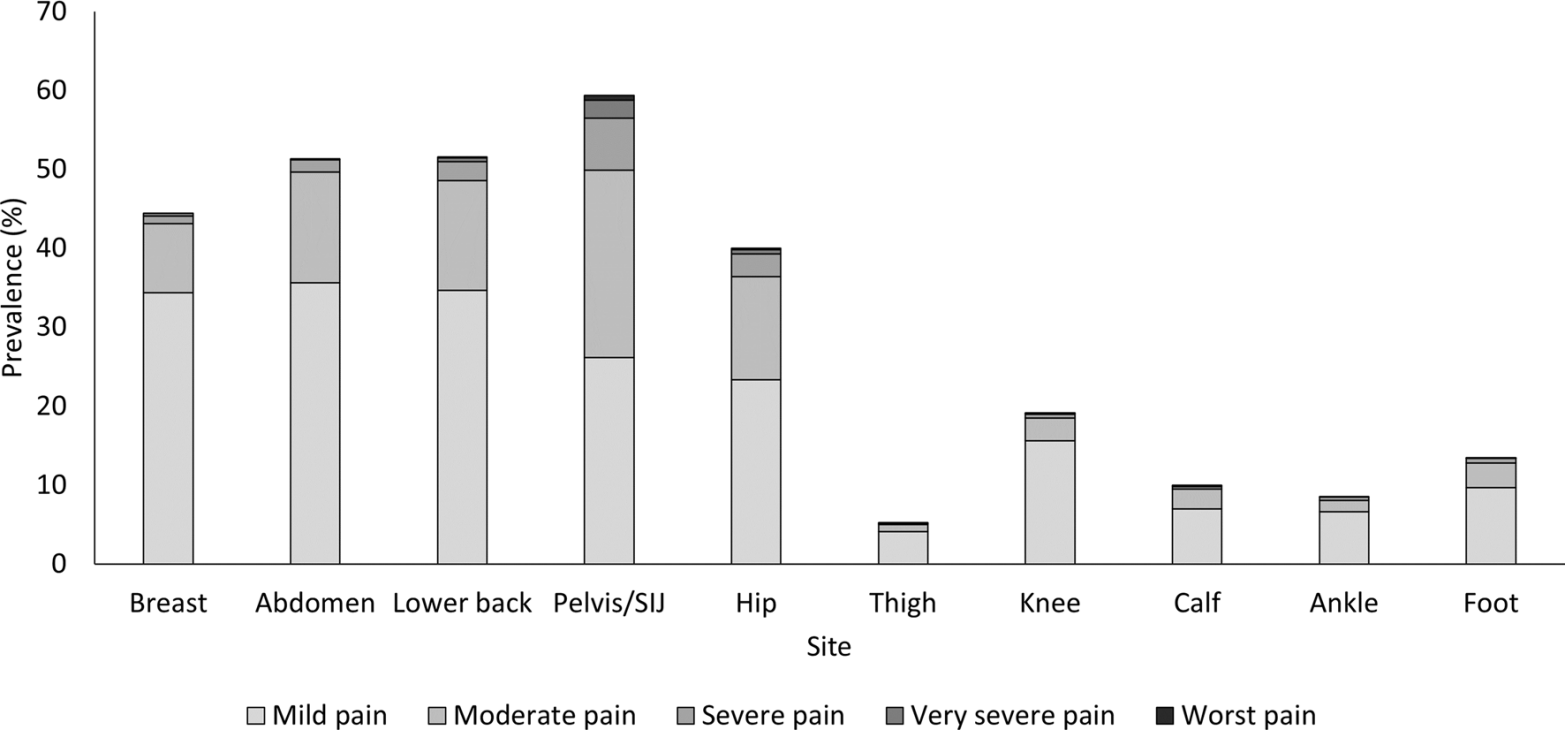
Pain prevalence rates (%) in 2667 women during-pregnancy across ten body sites. *SIJ* sacroiliac joint

Of the 3102 women, 2667 (86%) experienced pain while running during pregnancy. During-pregnancy pain prevalence was greatest for the pelvis/SIJ (59.4%), lower back (51.6%), abdomen (51.3%), breast (44.4%), hip (40.0%), knee (19.1%), foot (13.5%), calf (9.9%), ankle (8.5%) and thigh (5.2%) (Fig. 1 and Supplementary Table C in the electronic supplementary material). The pelvis/SIJ had the most prevalent moderate (23.7%), severe (6.6%), very severe (2.2%) and worst (0.6%) pain intensity ratings during pregnancy.

### Risk Factors for Musculoskeletal Pain when Running During Pregnancy

When all sites were combined, those at greatest risk of pain while running during pregnancy were those who had experienced a recurring pre-pregnancy injury (OR = 3.44, *p* < 0.001, 95% CI 2.05–5.78) and older women (OR = 1.04, *p* = 0.04, 95% CI 1.00–1.07). With each year of age, women experienced a 4% increased risk of pain while running during pregnancy. For example, a woman aged 35 is 20% and a woman aged 40 is 40% more likely to experience pain while running during pregnancy than a woman aged 30.

**Fig. 2:**
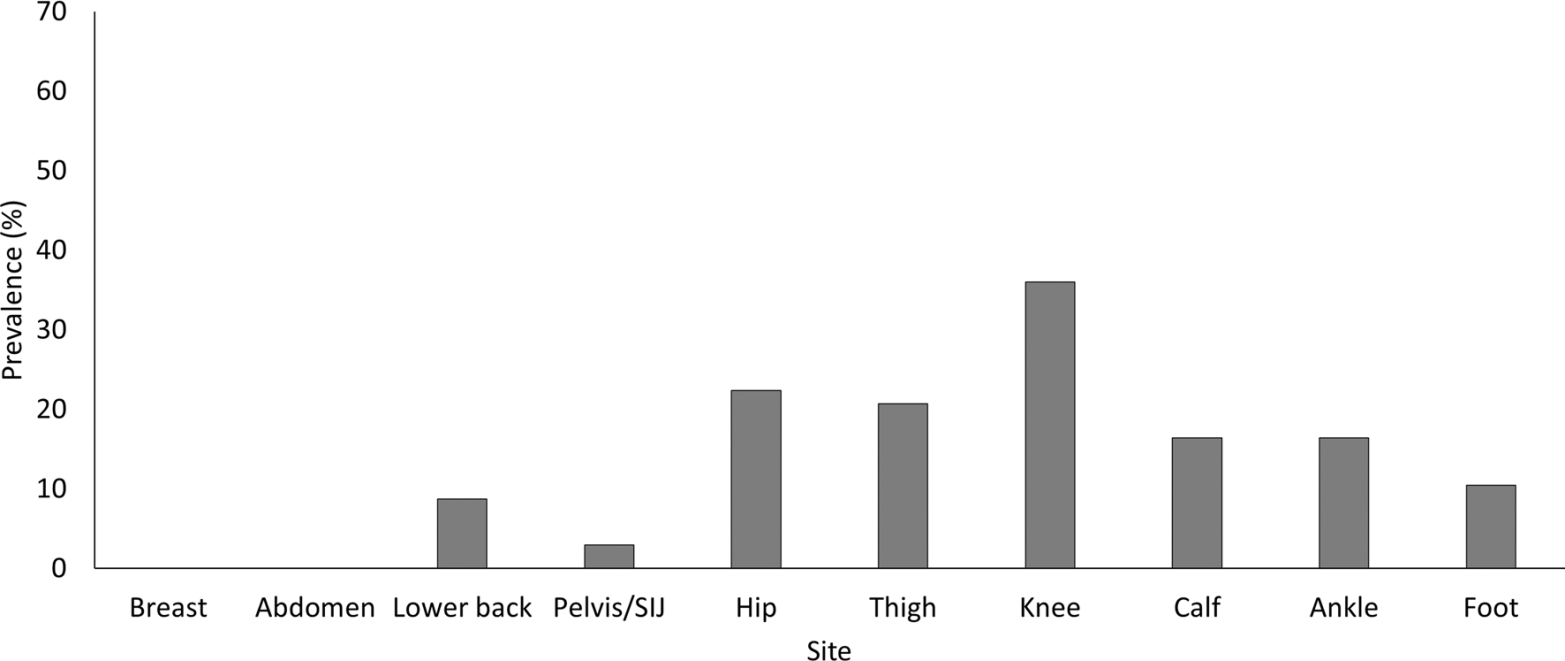
Recurring injury prevalence rates (%) in 459 women before pregnancy across ten body sites. *SIJ* sacroiliac joint

With increased age, women were more likely to experience thigh (OR = 1.07,* p* < 0.001, 95% CI 1.02–1.12), pelvis/SIJ (OR = 1.04,* p* < 0.001, 95% CI 1.02–1.06), knee (OR = 1.04,* p* < 0.001, 95% CI 1.01–1.07) and lower back (OR = 1.02,* p* = 0.04, 95% CI 1.00–1.04) pain. Recurring pre-pregnancy injuries were reported by 469 women (15.2%); the most common sites were the knee (36.0%), hip (22.4%), thigh (20.7%), ankle (16.4%), calf (16.4%), foot (10.4%), lower back (8.7%) and pelvis/SIJ (3.0%) (Fig. 2). Women who had experienced previous recurring injuries at any site were also more likely to experience pain at nine of the ten sites investigated (Table 2). Women with any recurring injury pre-pregnancy were more than two times more likely to experience pain in the lower limbs, i.e. knee (OR = 2.83,* p* < 0.001, 95% CI 2.18–3.68), ankle (OR = 2.18,* p* < 0.001, 95% CI 1.54–3.09) and calf (OR = 2.10,* p* < 0.001, 95% CI 1.50–2.95) during pregnancy. Of the 2868 women who completed questions on pre-pregnancy injury status and during-pregnancy pain, 73.3% reported during-pregnancy pain without having experienced recurring injuries pre-pregnancy. 15.3% experienced both injury pre-pregnancy and during-pregnancy pain, 10.7% experienced neither, and 0.8% experienced pre-pregnancy injury, but no during-pregnancy pain.

**Table 2:** Statistically significant predictors of pain experienced when running during pregnancy

Predictor (number of women)	Pain site	*P*	OR	95% CI
Had recurring pre-pregnancy injury (2957)	Knee	< 0.001	2.83	2.18–3.68
	Ankle	< 0.001	2.18	1.54–3.09
	Calf	< 0.001	2.1	1.50–2.95
	Hip	< 0.001	1.86	1.48–2.33
	Pelvis/SIJ	< 0.001	1.69	1.34–2.14
	Foot	< 0.001	1.69	1.25–2.29
	Breast	< 0.001	1.44	1.15–1.79
	Abdominal	< 0.001	1.39	1.11–1.75
	Lower back	0.02	1.31	1.04–1.64
Had previous children (3085)	Pelvis/SIJ	< 0.001	1.51	1.27–1.79
	Hip	< 0.001	1.38	1.16–1.65
	Knee	0.03	1.28	1.03–1.61
	Lower back	0.01	1.27	1.07–1.50
	Breast	< 0.001	0.76	0.64–0.90
Increased age (years) (2870)	Thigh	< 0.001	1.07^a^	1.02–1.12
	Pelvis/SIJ	< 0.001	1.04^a^	1.02–1.06
	Knee	< 0.001	1.04^a^	1.01–1.07
	Lower back	0.04	1.02^a^	1.00–1.04
Increased gestation of pregnancy-running cessation (weeks) (3033)	Calf	< 0.001	1.04	1.02–1.06
	Ankle	0.01	1.03	1.01–1.05
	Foot	0.01	1.02	1.00–1.04
Decreased gestation of pregnancy-running cessation (weeks) (3033)	Breast	< 0.001	1.02^a^	1.01–1.03
	Pelvis/SIJ	0.01	1.01^a^	1.00–1.02
Increased pre-pregnancy running experience (years) (3054)	Pelvis/SIJ	0.04	1.02	1.00–1.03
Decreased pre-pregnancy running experience (years) (3054)	Knee	0.01	1.03^a^	1.01–1.05
Decreased pre-pregnancy running frequency (times/week) (3012)	Knee	< 0.001	1.01^a^	1.01–1.02

*CI* confidence interval, *OR* odds ratio, *SIJ* sacroiliac joint

^a^Indicates OR was calculated as 1- logistic regression coefficient *β* to support interpretation; corresponding 95% CI values have additionally been amended to support interpretation

Women who had previous children were more likely to experience pain at the pelvis/SIJ (OR = 1.51,* p* < 0.001, 95% CI 1.27–1.79), hip (OR = 1.38,* p* < 0.001, 95% CI 1.16–1.65), knee (OR = 1.28,* p* = 0.03, 95% CI 1.03–1.61) and lower back (OR = 1.26,* p* = 0.01, 95% CI 1.07–1.50); however, they were less likely to experience breast pain (OR = 0.76,* p* < 0.001, 95% CI 0.64–0.90). Women had increased odds of knee pain if they were newer to running prior to pregnancy (OR = 1.03,* p* = 0.01, 95% CI 1.01–1.05) or ran less frequently pre-pregnancy (OR = 1.01,* p* < 0.001, 95% CI 1.01–1.02). Women who were more experienced prior to their pregnancy were more likely to experience pelvic/SIJ pain (OR = 1.02,* p* = 0.04, 95% CI 1.00–1.03). Pre-pregnancy running distance was not associated with an increased risk of pain while running during pregnancy. For each week run during pregnancy, women were more likely to experience calf (OR = 1.04,* p* < 0.001, 95% CI 1.02–1.06), ankle (OR = 1.03,* p* = 0.01, 95% CI 1.01–1.05) or foot (OR = 1.02,* p* = 0.01, 95% CI 1.00–1.04) pain. Additionally, women who stopped running earlier in pregnancy were more likely to experience breast (OR = 1.02,* p* < 0.001, 95% CI 1.01–1.03) or pelvic/SIJ (OR = 1.01,* p* = 0.01, 95% CI 1.00–1.02) pain. All logistic regression outputs are provided in Supplementary Table D (see the electronic supplementary material).

## Discussion

The study aimed to develop insight into musculoskeletal pain experienced by women when running during pregnancy. Informed by a diverse cohort of women who ran during any part of their pregnancy, the study findings offer novel insights that may be of interest to those who wish to run themselves or practitioners aiming to facilitate and support running during pregnancy.

### Running Habits

Many women aim to continue running during pregnancy; however, participation has been reported to decrease as pregnancy progresses, with only 31% of competitive runners continuing to run during their third trimester [6]. The women surveyed in the current study reduced their mean running distance compared to pre-pregnancy by 23%, 36% and 45% from the first to third trimester, respectively. This progressive reduction in training volume throughout pregnancy is consistent with that documented elsewhere in pregnant elite runners (45%, 50% and 70%), when considering their mean pre-pregnancy running volumes (elite: 114 km/week vs diverse survey cohort: 23.5 km/week) [27]. Progressive decreases in frequency, intensity and time of cardiovascular and resistance training exercise of 49%, 72% and 80% across trimesters have been previously documented [28]. Guidelines for physical activity during pregnancy encourage the accumulation of physical activity over a minimum of 3 days [4], which was the average number of runs our participants completed pre-pregnancy, and across the two first trimesters of pregnancy in those who continued to run. As reported in this survey, women do continue to run throughout their pregnancy, and in many cases (*n* = 223, 7.2%), women run up to the week they give birth. Healthcare professionals may support pregnant women who wish to run during their pregnancy by managing their expectations of running volume, and noting that a reduction in running distance and frequency is common as pregnancy progresses.

### During-Pregnancy Pain

Despite running shorter distances and less often compared to pre-pregnancy, 86% of the studied cohort reported experiencing pain while running during pregnancy. Pain was most prevalent at the pelvis/SIJ, lower back, abdomen, breast and hip (prevalence at each listed site ≥ 39.5%), with the most prevalent severe pain experienced at the pelvis/SIJ. It is of note that the sites of greatest pain prevalence are within body regions that undergo the greatest anatomical changes during pregnancy. As pain is one of the main perceived barriers to training during pregnancy [28], regions of substantial structural change require greater attention to support women’s ability to continue running through pregnancy. Further investigation of the usefulness of interventions such as working with a physiotherapist or other health professional, the type of breast support and the use of belly banding for pain reduction during running is warranted.

Importantly, pain during pregnancy has been identified as a risk factor for pain in postpartum runners [29], demonstrating potential long-term consequences of high during-pregnancy prevalence rates. Musculoskeletal pain prevalence rates during pregnancy from the current study (86%) aligned with postpartum runners studied previously (84%) [30]. In contrast to our current study findings, which exposed proximal body sites to be of most concern for pain, distal sites, specifically the lower limbs, have been found to be the regions of greatest musculoskeletal pain in a postpartum cohort [30]. Further research investigating the relationship between during-pregnancy pain and postpartum pain is needed to progress current understanding to support women’s engagement with running during this time of substantial physical change.

### Risk Factors for Musculoskeletal Pain when Running During Pregnancy

Approximately 40% of surveyed women experienced breast pain when running during pregnancy. A novel finding of the current study was that women who had a previous child were at significantly lower odds of experiencing breast pain while running during their subsequent pregnancies. During pregnancy, the breasts undergo structural changes [31], becoming larger and often warranting a revision of breast support [32]. During pregnancy, a complete remodelling of breast tissues occurs that does not return to the pre-pregnancy state post-lactation [31], likely accounting for the reduced odds of experiencing breast pain in subsequent pregnancies. Ensuring adequate breast support during running, particularly for women in their first pregnancies, may help reduce instances of pain, as has been found for women with larger breasts [32]. Providing targeted recommendations to pregnant runners regarding breast support is challenging given the absence of research on breast biomechanics of pregnant women [33]. Further research into breast biomechanics while running during pregnancy is required to inform practices. The experience of breast pain may not only limit running during pregnancy, but it is also a perceived barrier to returning to running postpartum [28].

Although having a previous child reduced the risk of experiencing breast pain, the odds of experiencing pain at the pelvis/SIJ, hip, knee and lower back were greater (OR ≥ 1.26) for those who had previous children. Previous findings have demonstrated altered gait mechanics postpartum compared with nulliparous controls [34]. Our observations of increased odds of running pain while pregnant with successive pregnancies may therefore, in part, be a consequence of altered running gait mechanics. Associations have additionally been evidenced between altered gait mechanics and pain in nulliparous populations [35, 36]. Although it is challenging to conduct longitudinal studies in pregnant women, analysis of running gait mechanics and pain experienced across successive pregnancies would provide valuable insight to inform approaches for pain prevention.

Those with any recurring pre-pregnancy injuries had over two times more risk of experiencing lower-limb pain during pregnancy, specifically at the knee, ankle and calf. Our findings align with research from nulliparous populations, which reports previous injury as a main running-related injury risk factor [19, 37], with running injuries predominantly located in the lower limbs [13]. Although to a lesser extent, previous recurring injuries also played a role in increasing the odds of experiencing pain at the hip, pelvis/SIJ, foot, breast, abdominal region and lower back during pregnancy in the women surveyed. Details of pre-pregnancy injury, and the extent to which the runners had recovered from those injuries prior to running during pregnancy, were not measured within the current study. These factors are important to further investigate to progress current understanding of factors associated with pre-pregnancy running-related injury [38].

Women who stopped running earlier in their pregnancy presented with greater odds of pelvic/SIJ and breast pain, suggesting pain experienced at these sites was the greatest musculoskeletal barrier to continuing to run during pregnancy. Efforts to introduce pain prevention, reduction and management strategies (e.g. gait training to reduce joint loads [37] and improved breast support [32]) should place emphasis on the pelvis and breasts. Those with more pre-pregnancy running experience were additionally at greater risk of pelvic/SIJ pain, whereas those with less running experience before becoming pregnant were more likely to experience knee pain while running during their pregnancy. As within a nulliparous population, less experienced runners tend to have a greater rate of lower-limb injuries [39]. For novice runners, a progressive running approach is recommended to allow anatomical adjustments to running stresses [38]. Sharing this information with women who are trying to become pregnant, or who are early in their pregnancy, may support them to be able to continue to run during pregnancy, should they wish to.

### Strengths and Limitations of the Study

A prominent strength of this study was the international cohort of women who shared their pre- and during-pregnancy running experiences. Our study cohort had a broad range of ages (34 ± 3.9 years; 22–52 years) and running experience (8.6 ± 6.0 years; 0.5–35 years), albeit with a high proportion of experienced runners (72.5%). Data were collected from December 2021 to March 2022; subsequently, many of the participants will have reported on pregnancies during the coronavirus disease 2019 (COVID-19) period. It is likely that restrictions on events, outdoor activity, gym access, etc. will have disrupted the typical running behaviour of many participants. Like all studies involving the recruitment of volunteers, the study was subject to non-response bias, which could have impacted the degree to which the samples accurately represent the wider population of women who ran during pregnancy.

The current study was retrospective, and the ability to recall pain along with other running habits and personal details (e.g. pre-pregnancy injury) was likely impacted by the passage of time. While the recall period was purposely limited to 5 years, with a cohort mean of 1.9 years, it is likely that the accuracy of responses was impacted by the retrospective nature of the study and the length of the recall period. To limit recall bias, future prospective studies are recommended that follow guidelines [40] on methodology for recording overuse symptoms, including pain, in sports.

An additional limitation of the current study was the lack of data collected on pre-pregnancy pain and during-pregnancy injury. The decision to focus on during-pregnancy pain was purposeful, as outlined previously; however, additional data on whether pain translated to an injury during pregnancy would have been of additional interest. Similarly, the decision to include pre-pregnancy injury as a personal risk factor was made in line with previous research; however, knowledge of pre-pregnancy pain during running would have also been of interest to expand the current analysis. The relationship between pain and injury in running requires more attention in future research.

The current survey did not encompass all musculoskeletal issues experienced by women who run during pregnancy. It is well understood that pelvic health issues often play a significant role in women’s physical experiences during pregnancy. Incontinence and pelvic floor issues were not considered within the current article; however, research in the respective areas is important to contribute to the knowledge supporting women who wish to run during pregnancy.

## Conclusions

Based on self-reported experiences of over 3000 women, running distances and frequency were reduced at each trimester compared to pre-pregnancy. Pain while running during pregnancy was common (86%), and most likely experienced at the pelvis/SIJ, lower back, abdomen, breasts and hips. Those at greater odds of experiencing pain (at any site) reported a pre-pregnancy recurring injury or were older. Women who had a previous child were less likely to experience breast pain than those running in their first pregnancy, but more likely to experience pain at the pelvis, hips, knees and lower back. Pelvis and breast pain were the greatest musculoskeletal barriers to the continuation of running during pregnancy; therefore, pain prevention strategies should prioritise these body sites. The study findings may help inform healthcare education to support women to be able to engage in running during pregnancy.

### Supplementary Information

Below is the link to the electronic supplementary material.

Supplementary file1 (PDF 300 KB)

Supplementary file2 (DOCX 28 KB)

## Footnotes

1 ‘Woman’ or ‘women’ are used to refer to the pregnant and birthing person. These designations were selected to avoid confusion, as the literature and the most recent Ministry of Health of New Zealand documents also use mother and woman [1]. It is acknowledged that not all pregnant and birthing people identify as a woman or mother.

## Declarations

### Data Availability

Data are available from the lead author on request.
