# Supplementary material for: Prevalence and Risk Factors for Musculoskeletal Pain when Running During Pregnancy: A Survey of 3102 Women
Source: Sports Med. 2024 Feb 6;54(7):1955–64. doi: 10.1007/s40279-024-01994-6 (PMC11258093; doi:10.1007/s40279-024-01994-6)
Supplement: Supplementary file 1 — Supplementary file1 (PDF 300 KB) [file 40279_2024_1994_MOESM1_ESM.pdf]

Sports Medicine

Prevalence and risk factors for musculoskeletal pain when running during pregnancy: a survey of 3,102 women

Short title/running head: Pain when running during pregnancy

Hannah E Wyatt[1,2*], Kelly Sheerin[2], Patria A Hume[2,3,4], Kim Hébert-Losier[5]
[1]Faculty of Health, University of Canterbury, Christchurch, New Zealand
[2]Sports Performance Research Institute New Zealand, Auckland University of Technology, Auckland, New Zealand
[3]Auckland Bioengineering Institute, The University of Auckland, Auckland, New Zealand
[4]Mindaroo Tech & Policy Lab, Law School, The University of Western Australia, Perth, Australia
[5]Division of Health, Engineering, Computing and Science, Te Huataki Waiora School of Health University of Waikato, Tauranga, New Zealand

Supplementary Information: Social media advertisement.

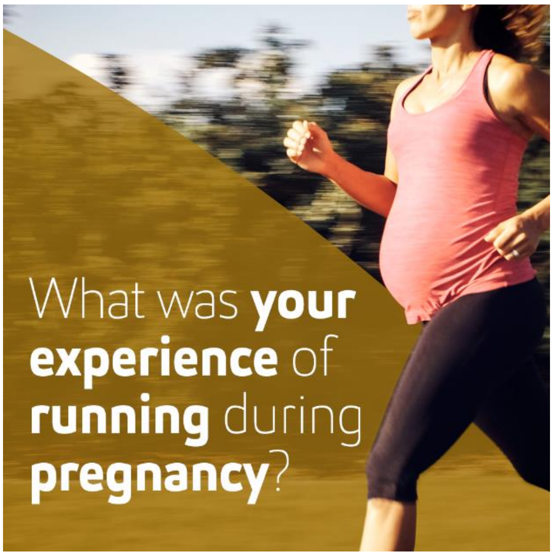

Accompanying text:
Help us develop knowledge of running during pregnancy by sharing your experience...
If you:

- ✓ Have a child under 5 years of age
- ✓ Ran before and during (any of) your pregnancy

...take our 10 minute survey:
https://aut.au1.qualtrics.com/jfe/form/SV_0ufJ01nOD6WBBoW
Please share with your networks to help us in our quest to improve women's activity during their pregnancy

Supplementary Information: Survey questions

A survey of women's experiences of running during pregnancy - Participant Information

Thank you for considering this invitation to participate in our research. We are researchers at Auckland University of Technology (AUT) who are interested in studying physical changes through pregnancy and their influence on movement and activity. The current study involves a short survey (approximately 10-15 minutes).

The aim of the research is to develop insight into women's experiences of running during pregnancy, including running habits, musculoskeletal discomfort/injuries and the timing and reasons for having to change or stop running.

What are we trying to find out? Physical activities such as running are important for all individuals, including women who are pregnant. As women progress through pregnancy their bodies change in a way that can dramatically affect the way they move. For many, the desire to continue running can be tempered by discomfort and physical concerns. Our knowledge of these concerns would be highly valuable to inform health practices to support running during pregnancy. Knowledge is currently limited.

Who are those we wish to be involved? We invite all women who have had a child in the past 5 years, who ran during (any part of) their pregnancy, and who ran at least once per week for a minimum of 20 minutes prior to their pregnancy.

What would we like the participants to do? We invite you to complete a one-off short electronic survey about your experience of running during pregnancy.

How do you agree to participate in this research? Your participation in this research is voluntary (it is your choice) and whether or not you choose to participate will neither advantage nor disadvantage you. At the bottom of this page, you will be asked if you agree to participate or not.

What will happen in this research? If you choose to participate, you will be taken to the first page of the survey. In total, there are 26 questions which we will ask for you to complete, but you may leave out any you wish to. The survey takes approximately 10-15 minutes to complete. On completion you will be asked to submit the survey and the answers will be submitted to our research team. The submitted answers are anonymous, meaning we will not know who they have come from.

What are the benefits? The findings of this study will help us better understand the experiences of women who run during pregnancy. These insights will help direct further research to address the challenges identified in the survey.

How will your privacy be protected? All collected data will be anonymous therefore it will not be possible to link your responses back to you. All data will be stored securely for 10 years.

What are the costs of participating in this research? Other than your time, there is no cost to participate in this research.

What opportunity do I have to consider this invitation? The survey link will remain open for three months.

Will you receive feedback on the results of this research? The final results of the study will be

published in a peer-reviewed journal, however if you are interested in seeing a summary of these results, they will be available via this link in March 2022.

This study was approved by the Auckland University of Technology Ethics Committee (AUTEC) on November 11th, 2021 (AUTEC Reference 21/401).

Please confirm the following points before proceeding.

- I am over the age of 18 years of age.
- I have a child who is currently under the age of 5 years of age.
- I regularly ran at least 1 time per week for a minimum of 20 minutes before this pregnancy.
- I have read and understood the information above and consent to participate in this survey.

Section 1: Demographics

Q1.1: What is your current age?

Q1.2: What was your age at the birth of your youngest child?

Q1.3: What is your ethnicity?
- British
- Australian
- European Other
- African
- New Zealand European/Pakeha
- Irish
- Dutch
- Chinese
- Latin American
- Indian
- German
- Polish
- Italian
- French
- Spanish
- Middle Eastern
- Asian Other
- Māori
- Filipino
- Sri Lankan
- Cook Islands Māori
- Vietnamese
- Other Ethnicity
- Prefer not to say

Q1.4: How many children have you given birth to?

Q1.5: Which country did you live in during your pregnancy?

Section 2: Pre-pregnancy

Please answer the questions in this section in relation to the 6 months prior to the pregnancy of your youngest child (i.e. your latest birth).

Q2.1: In the 6 months before your latest pregnancy, did you class yourself as a novice, recreational or experienced runner?

- o Novice (Less than 6 months of running at least 1 per week)
- o Recreational (Between 6 months and 3 years of running at least 1 per week)
- o Experienced (More than 3 years of running at least 1 per week)
- o I don't recall

Q2.2: Before your pregnancy, how many years had you been running regularly?

Q2.3: In the 6 months before your latest pregnancy, on average, how far would you run each week?

- o Km
- o Miles
- o I don't recall

Q2.4: In the 6 months before your latest pregnancy, how many times would you run each week? [Select one]

- o 1
- o 2
- o 3
- o 4
- o 5
- o 6
- o 7+
- o I don't recall

Q2.5: Did you have any recurring running injuries before your last pregnancy?

- o Yes
- o No
- o I don't recall

Q2.6: What were your recurring running injuries? [Please provide details]

Section 3: During-pregnancy

Please answer the remaining questions in relation to your youngest child.

Q3.1: Did your last pregnancy result in a single or multiple birth?

- o Single
- o Multiple (please specify)

Q3.2: How many weeks pregnant were you when you gave birth?

Q3.3: Did you run while you were pregnant? [Note: the remainder of this survey is directed toward women who ran during their pregnancy, if you did not, the survey will end.]

- o Yes
- o No

Q3.4: How many weeks through your pregnancy were you when you stopped running?

Q3.5: During each trimester of your pregnancy, approximately how far (distance) did you run each week?

Trimester 1 (Weeks 1-13)

- Km
- Miles
- I don't recall

Trimester 2 (Weeks 14-27)

- Km
- Miles
- I don't recall

Trimester 3 (Weeks 28-40)

- Km
- Miles
- I don't recall

Q3.6: During each trimester of your pregnancy, approximately how many times would you run each week? [Select all that apply]

- Trimester 1 (Weeks 1-13)
- Trimester 2 (Weeks 14-27)
- Trimester 3 (Weeks 28-40)

Q3.7: On the scale below please report any pain you experienced when you ran while pregnant?

	No pain	Mild pain	Moderate pain	Severe pain	Very severe pain	Worst pain possible
Breast						
Abdominal						
Lower back						
Pelvis/Sacroiliac joint						
Hip						
Thigh						
Knee						
Calf						
Ankle						
Foot						
