# Supplementary material for: Prevalence and Risk Factors for Musculoskeletal Pain when Running During Pregnancy: A Survey of 3102 Women
Source: Sports Med. 2024 Feb 6;54(7):1955–64. doi: 10.1007/s40279-024-01994-6 (PMC11258093; doi:10.1007/s40279-024-01994-6)
Supplement: Supplementary file 2 — Supplementary file2 (DOCX 28 KB) [file 40279_2024_1994_MOESM2_ESM.docx]

**Sports Medicine**

**Prevalence and risk factors for musculoskeletal pain when running during pregnancy: a survey of 3,102 women**

**Short title/running head: Pain when running during pregnancy**

**Hannah E Wyatt1,2*, Kelly Sheerin2, Patria A Hume2,3,4, Kim Hébert-Losier5**

1Faculty of Health, University of Canterbury, Christchurch, New Zealand

2Sports Performance Research Institute New Zealand, Auckland University of Technology, Auckland, New Zealand

3Auckland Bioengineering Institute, The University of Auckland, Auckland, New Zealand

4Mindaroo Tech & Policy Lab, Law School, The University of Western Australia, Perth, Australia

## 5Division of Health, Engineering, Computing and Science, Te Huataki Waiora School of Health University of Waikato, Tauranga, New Zealand

## Supplementary Table A. Ethnicity of the 3,081 respondents to the questions.

| **Ethnicity** | **Count (*n*)** | **% participants** |
| --- | --- | --- |
| British | 1917 | 62.2 |
| Australian | 363 | 11.8 |
| European Other | 172 | 5.6 |
| African | 102 | 3.3 |
| New Zealand European/Pakeha | 97 | 3.1 |
| Irish | 81 | 2.6 |
| Dutch | 32 | 1.0 |
| Chinese | 19 | 0.6 |
| Latin American | 18 | 0.6 |
| Indian | 17 | 0.6 |
| German | 16 | 0.5 |
| Polish | 14 | 0.5 |
| Italian | 13 | 0.4 |
| French | 9 | 0.3 |
| Spanish | 7 | 0.2 |
| Middle Eastern | 5 | 0.2 |
| Asian Other | 5 | 0.2 |
| Māori | 3 | 0.1 |
| Filipino | 3 | 0.1 |
| Sri Lankan | 2 | 0.1 |
| Cook Islands Māori | 1 | 0.0 |
| Vietnamese | 1 | 0.0 |
| Other Ethnicity | 136 | 4.4 |
| Prefer not to say | 48 | 1.6 |

# Supplementary Table B. Countries lived in during pregnancy of the 3,095 respondents to the questions.

| **Country** | **Count (*n*)** | **% participants** |
| --- | --- | --- |
| United Kingdom/Ireland | 2013 | 65.0 |
| Australia | 437 | 14.1 |
| South Africa | 252 | 8.1 |
| United States of America | 193 | 6.2 |
| New Zealand | 122 | 3.9 |
| Canada | 43 | 1.4 |
| France | 5 | 0.2 |
| United Arab Emirates | 4 | 0.1 |
| Hong Kong | 3 | 0.1 |
| Netherlands | 3 | 0.1 |
| Switzerland | 3 | 0.1 |
| Germany | 2 | 0.1 |
| Japan | 2 | 0.1 |
| Mexico | 2 | 0.1 |
| Austria | 1 | 0.0 |
| Azerbaijan | 1 | 0.0 |
| Belgium | 1 | 0.0 |
| China | 1 | 0.0 |
| Denmark | 1 | 0.0 |
| Ecuador | 1 | 0.0 |
| Georgia | 1 | 0.0 |
| Israel | 1 | 0.0 |
| Qatar | 1 | 0.0 |
| Sweden | 1 | 0.0 |
| Zimbabwe | 1 | 0.0 |

## Supplementary Table C. Pain (% prevalence) experienced across 10 body sites during pregnancy for the 3,102 respondents to the questions.

| **Body site** | **Mild** | **Moderate** | **Severe** | **Very severe** | **Worst** | **Any pain** |
| --- | --- | --- | --- | --- | --- | --- |
| **Breast** | 34.3 | 8.7 | 1.0 | 0.4 | 0.0 | 44.4 |
| **Abdominal** | 35.6 | 14.1 | 1.5 | 0.0 | 0.1 | 51.3 |
| **Lower back** | 34.6 | 13.9 | 2.4 | 0.5 | 0.1 | 51.6 |
| **Pelvis/SIJ** | 26.1 | 23.7 | 6.6 | 2.2 | 0.6 | 59.4 |
| **Hip** | 23.4 | 13.0 | 2.9 | 0.6 | 0.2 | 40.0 |
| **Thigh** | 4.1 | 0.9 | 0.1 | 0.1 | 0.0 | 5.2 |
| **Knee** | 15.6 | 2.9 | 0.4 | 0.1 | 0.0 | 19.1 |
| **Calf** | 7.0 | 2.5 | 0.3 | 0.1 | 0.0 | 9.9 |
| **Ankle** | 6.6 | 1.4 | 0.4 | 0.0 | 0.0 | 8.5 |
| **Foot** | 9.7 | 3.1 | 0.6 | 0.0 | 0.0 | 13.5 |

Note: SIJ=sacroiliac joint.

## Supplementary Table D. Predictors of pain for all body sites.

| **Predictor** (number of women) | **Pain site** | ***p*** | **OR** | **95% CI (lower)** |
| --- | --- | --- | --- | --- |
| Had recurring pre-pregnancy injuries (2,957) | Breast | <0.001 | 1.44 | 1.15-1.79 |
|  | Abdominal | <0.001 | 1.39 | 1.11-1.75 |
|  | Lower back | 0.02 | 1.31 | 1.04-1.64 |
|  | Hip | <0.001 | 1.86 | 1.48-2.33 |
|  | Thigh | 0.37 | 1.24 | 0.77-2.02 |
|  | Pelvis/SIJ | <0.001 | 1.69 | 1.34-2.14 |
|  | Knee | <0.001 | 2.83 | 2.18-3.68 |
|  | Calf | <0.001 | 2.1 | 1.50-2.95 |
|  | Ankle | <0.001 | 2.18 | 1.54-3.09 |
|  | Foot | <0.001 | 1.69 | 1.25-2.29 |
| Had previous children (3,085) | Breast | <0.001 | 0.76 | 0.64-0.90 |
|  | Abdominal | 0.49 | 0.94 | 0.80-1.11 |
|  | Lower back | 0.01 | 1.27 | 1.07-1.50 |
|  | Hip | <0.001 | 1.38 | 1.16-1.65 |
|  | Thigh | 0.22 | 1.26 | 0.87-1.84 |
|  | Pelvis/SIJ | <0.001 | 1.51 | 1.27-1.79 |
|  | Knee | 0.03 | 1.28 | 1.03-1.61 |
|  | Calf | 0.74 | 1.05 | 0.78-1.41 |
|  | Ankle | 0.26 | 0.84 | 0.61-1.14 |
|  | Foot | 0.75 | 0.96 | 0.75-1.24 |
| Increased age (years) (2,870) | Breast | 0.45 | 1.01 | 0.99-1.03 |
|  | Abdominal | 0.11 | 1.02 | 1.00-1.04 |
|  | Lower back | 0.04 | 1.02 | 1.00-1.04 |
|  | Hip | 0.06 | 1.02 | 1.00-1.04 |
|  | Thigh | <0.001 | 1.07 | 1.02-1.12 |
|  | Pelvis/SIJ | <0.001 | 1.04 | 1.02-1.06 |
|  | Knee | <0.001 | 1.04 | 1.01-1.07 |
|  | Calf | 0.12 | 1.03 | 0.99-1.07 |
|  | Ankle | 0.28 | 1.02 | 0.98-1.06 |
|  | Foot | 0.68 | 1.01 | 0.97-1.04 |
| Increased gestation of pregnancy-running cessation (weeks) (3,033) | Abdominal | 0.13 | 1.01 | 1.00-1.02 |
|  | Lower back | 0.9 | 1.00 | 0.99-1.01 |
|  | Hip | 0.91 | 1.00 | 0.99-1.01 |
|  | Thigh | 0.09 | 1.02 | 1.00-1.05 |
|  | Knee | 0.96 | 1.00 | 0.99-1.01 |
|  | Calf | <0.001 | 1.04 | 1.02-1.06 |
|  | Ankle | 0.01 | 1.03 | 1.01-1.05 |
|  | Foot | 0.01 | 1.02 | 1.00-1.04 |
| Decreased gestation of pregnancy-running cessation (weeks) (3,033) | Breast | <0.001 | 1.02 | 1.01-1.03 |
|  | Pelvis/SIJ | 0.01 | 1.01 | 1.00-1.02 |
| Increased pre-pregnancy running experience (years) (3,054) | Breast | 0.94 | 1.00 | 0.99-1.02 |
|  | Abdominal | 0.34 | 1.01 | 0.99-1.02 |
|  | Pelvis/SIJ | 0.04 | 1.02 | 1.00-1.03 |
| Decreased pre-pregnancy running experience (years) (3,054) | Lower back | 0.43 | 1.01 | 0.99-1.02 |
|  | Hip | 0.28 | 1.01 | 0.99-1.02 |
|  | Thigh | 0.6 | 1.01 | 0.97-1.04 |
|  | Knee | 0.01 | 1.03 | 1.01-1.05 |
|  | Calf | 0.66 | 1.01 | 0.98-1.03 |
|  | Ankle | 0.32 | 1.01 | 0.99-1.04 |
|  | Foot | 0.73 | 1.00 | 0.98-1.03 |
| Increased pre-pregnancy running frequency (times/week) (3,012) | Abdominal | 0.53 | 1.00 | 1.00-1.01 |
|  | Lower back | 0.15 | 1.00 | 1.00-1.01 |
|  | Thigh | 0.64 | 1.00 | 0.99-1.01 |
|  | Pelvis/SIJ | 0.2 | 1.00 | 1.00-1.01 |
| Decreased pre-pregnancy running frequency (times/week) (3,012) | Breast | 0.07 | 1.00 | 1.00-1.01 |
|  | Hip | 0.21 | 1.00 | 1.00-1.01 |
|  | Knee | <0.001 | 1.01 | 1.01-1.02 |
|  | Calf | 0.64 | 1.00 | 0.99-1.01 |
|  | Ankle | 0.66 | 1.00 | 0.99-1.01 |
|  | Foot | 0.33 | 1.00 | 1.00-1.01 |

Note: SIJ=sacroiliac joint.
